# Supplementary figures and images for: Characterization, Molecular Mechanism of Prochloraz-Resistance in Fusarium fujikuroi and Development of Loop-Mediated Isothermal Amplification Rapid Detection Technique Based on the S312T Genotype of Resistances
Source: J Fungi (Basel). 2024 Aug 8;10(8):560. doi: 10.3390/jof10080560 (PMC11355441; doi:10.3390/jof10080560)

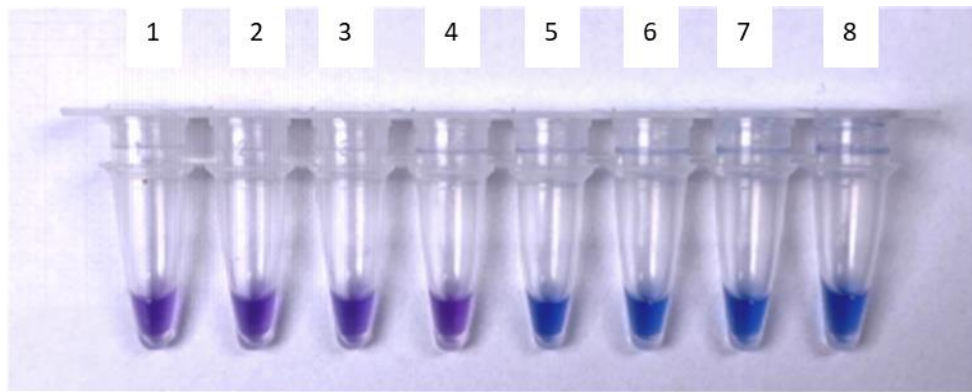

Figure S1 Detection of isolates collected in 2022

1-4 sensitive isolates: F5, F8, F 10, F25; 5-8 resistant isolates: F1, F54, F68, F72.

Supplement: Supplementary file 1 [file jof-10-00560-s001.zip › jof-3087282-supplementary.pdf]
